# Impact of Demographic Factors and Other Characteristics on Housing Outcomes at Discharge from Transitional Care Program

**DOI:** 10.3390/ijerph23060749

**Published:** 2026-06-03

**Authors:** Kayla R. Whitebrook, Mina Silberberg, Sandra Stinnett, Donna Biederman

**Affiliations:** 1 School of Medicine, Duke University, Durham, NC 27710, USA; 2 Department of Family Medicine and Community Health, Duke University, Durham, NC 27710, USA; mina.silberberg@duke.edu; 3 Department of Biostatistics and Bioinformatics, Duke University, Durham, NC 27710, USA; sandra.stinnett@duke.edu; 4 Department of Nursing, Duke University, Durham, NC 27710, USA; donna.biederman@duke.edu

**Keywords:** homelessness, housing, transitional care program

## Abstract

**Highlights:**

**Public health relevance—How does this work relate to a public health issue?**

- Homelessness is a significant public health concern in the United States given the large numbers of Americans experiencing homelessness and the association of homelessness with poor health outcomes.
- Transitional care programs have been shown to help some people experiencing homelessness obtain housing with associated positive impacts on health outcomes.

**Public health significance—Why is this work of significance to public health?**

- Despite homelessness being a significant public health concern, knowledge about which specific factors make PEH more likely to become rehoused is limited.
- Having a greater understanding of what factors are associated with an increased likelihood of rehousing could ultimately contribute to improved rehousing of people experiencing homelessness.

**Public health implications—What are the key implications or messages for practitioners, policy makers and/or researchers in public health?**

- Our study found that the odds of being rehoused as a non-White person are significantly less than the odds of being rehoused as a White person; with a greater understanding and awareness around these racial differences in rehousing PEH, more effective and targeted housing interventions could be created and employed to achieve more equitable rehousing.
- A novel contribution to the literature from our study is that having more unmet identification and communication access needs makes people experiencing homelessness less likely to be rehoused; if the evidence continues to support the importance of meeting these needs, then it would be appropriate for transitional care programs and other interventions for this population to prioritize meeting these needs more effectively so as to increase the likelihood of people experiencing homelessness being rehoused.

**Abstract:**

Homelessness is a major public health concern, and successful rehousing is an important outcome for people experiencing homelessness (PEH). However, limited evidence exists on which individual factors are associated with rehousing after transitional care; this study examined characteristics associated with being rehoused at discharge from Durham Homeless Care Transitions (DHCT). We analyzed data from DHCT, a transitional care program serving PEH. Independent variables included demographic characteristics, self-efficacy, mental healthcare status, and unmet identity and communication access (ICA) needs, including lack of personal identification documentation and technology access. We performed bivariate analyses and multivariable regression to assess associations with being rehoused at discharge. In both bivariate and multivariable analyses, non-White PEH were less likely to be rehoused at discharge than White PEH. Greater unmet ICA needs were also significantly associated with lower likelihood of rehousing. These findings add to the mixed prior literature regarding racial inequities in rehousing among PEH. The concept of ICA needs has not previously been studied and may offer an actionable target for transitional care programs seeking to improve rehousing outcomes and advance equity.

## 1. Introduction

Homelessness is a significant public health concern in the United States; large numbers of Americans are experiencing homelessness at any given time [1] and homelessness is associated with poor health outcomes [2]. Transitional care programs have been shown to help some people experiencing homelessness (PEH) obtain housing. Understanding who these programs work best for would help homeless service agencies to shape services in a way that could improve outcomes for PEH exiting such programs [3,4,5,6]. However, knowledge about specific participant factors associated with exiting homelessness is quite limited and knowledge of factors associated with success in leaving transitional programs for housing is even more limited. In this paper, using Durham Homeless Care Transitions’ transitional program administrative data, we describe the association of baseline client factors with the likelihood of being housed upon discharge from the program.

Medical respite programs began in the mid-1980s to help PEH with ongoing medical problems by providing a safe place to recover from illness and injury [7]. Many also offer interdisciplinary case management services, including helping people find housing [8,9]. These programs are sometimes referred to as transitional care programs. Some programs demonstrated improved housing status for PEH upon program exit [3,4,5,6]. There is substantial evidence about what factors may make a person more likely to become homeless, but there is less research on demographic factors and other individual characteristics that increase the likelihood of PEH becoming rehoused, with almost none focusing on which specific factors increase the likelihood of PEH becoming rehoused after medical respite or transitional care program engagement.

Because some transitional care programs are tailored to meet individual needs, having a greater understanding of what factors are associated with an increased likelihood of rehousing may offer insight to improve programmatic capacity to meet PEH’s need for improved housing, a notion shared by the existing literature exploring similar themes [10].

Education and gender have well-supported effects on the likelihood of PEH becoming rehoused; however, the evidence on the impact of age, race, and mental healthcare is much more heterogenous. People experiencing homelessness with higher education are more likely to exit homelessness than those with less education [11,12]. Female PEH have consistently been shown to have a higher likelihood of exiting homelessness compared to male PEH [13,14]. Evidence on the effect of age and race is mixed. For instance, there is some evidence that older PEH have more difficulty being rehoused [15,16], but other evidence suggests that age is not a factor [10]. Most prior research has shown that Black people are less likely to exit homelessness than White people [17,18,19], though one study has shown there is no difference in likelihood of PEH being rehoused by race [12]. Research on the impact of the presence of mental illness on the likelihood of exiting homelessness also has mixed results [12,20,21,22,23]. It is known that PEH have difficulty accessing mental health services [24], but research on whether access to mental health services for PEH improves likelihood of being rehoused is also mixed [25,26,27,28]. Furthermore, there is no research regarding whether the odds of being rehoused are different for PEH with mental illness who access mental health services prior to utilizing a transitional care program.

There is also overall little research on the effect of other characteristics associated with personal capacity on rehousing. While there is evidence that higher perceived self-efficacy may be associated with PEH being rehoused [13,29], that evidence is minimal and dated. There is one study indicating that lacking identification documentation is a barrier to being rehoused [30], and the ability to find and contact social services necessitates digital access and digital communication [31], indicating that lack of cell phone or email access might also be a barrier to being rehoused. However, there is no additional research about need burden—in the context of personal identification documentation and technology access—and its direct impact on PEH being rehoused.

Our primary goals for this study were to use an administrative data set from Durham Homeless Care Transitions (DHCT), a transitional care program, to: (1) describe enrolled participants, (2) report on the percentage of participants housed on discharge, and (3) identify individual characteristics associated with likelihood of being housed upon discharge to further clarify and contribute to knowledge in this area. The research questions that guided this study were:

1. What is the demographic distribution of participants in our transitional care program including age, race, gender, and education?
2. What is the distribution of self-perceived self-efficacy scores among participants?
3. What is the unmet ICA need burden and mental healthcare status of participants upon enrollment?
4. What percentage of participants are housed upon discharge?
5. Do any of the identified individual characteristics predict a higher likelihood of being housed upon discharge from the program?

Based largely on the existing literature [11,12,13,14,15,16,17,18,19,20,21,22,23,24,25,26,27,28,29,30,31], we hypothesized that PEH who are younger, female, White, have higher levels of educational attainment, and who do not have nor need mental healthcare would be more likely to be rehoused after transitional care program engagement. Based on our understanding of self-efficacy as a facilitating factor for positive outcomes in general and unmet needs as a barrier, we also hypothesized that PEH who have higher self-efficacy and fewer unmet ICA needs would be more likely to be rehoused.

## 2. Materials and Methods

### 2.1. Study Design

Using the DHCT program administrative dataset comprising data collected from intake information instruments including a demographic-focused enrollment form, a self-efficacy rating scale, and a has/needs inventory form, we conducted a retrospective cohort study to identify factors associated with likelihood of being housed upon discharge from the program.

### 2.2. Setting and Participants

The study setting is the DHCT Program, located in Durham, North Carolina and administered by Project Access of Durham County, a non-profit organization dedicated to improving healthcare for the uninsured and underinsured. DHCT is a transitional care program for PEH with acute or chronic medical issues with an ongoing medical need on discharge from the hospital; the program offers case consultation, care management, and medical respite services (when indicated).

This program was previously thoroughly described elsewhere [4]. DHCT began enrolling participants on 1 July 2016. Data from participants in this study were collected from enrollments beginning on 1 July 2016 through discharges on 31 December 2021. Eligibility criteria for DHCT include that the participant is able to participate in and maintain a safe and harm-free environment, is expected to follow rules of the housing setting (which may include maintenance of sobriety), is willing to participate in case management visits and a treatment plan, is competent in activities of daily living, and is psychiatrically stable. Program discharge occurs when the medical issue is stabilized or resolved or when the client is no longer able to live independently, relocates from the area, is incarcerated, becomes disengaged from the program, or dies. DHCT allows PEH to utilize the service more than once. Data for this study were compiled from the experiences of 142 of the total 187 participants enrolled in DHCT during the study window. A total of 44 of the excluded 45 participants were removed from the data set due to missing data, while the other participant was excluded because they would have created a cell size of one for the gender variable, as discussed further in the explanation of independent variables. In the case that a participant had more than one period of enrollment in DHCT, only data from the participant’s initial experience at DHCT were included in the study.

### 2.3. Data Collection

The DHCT Program programmatic data are housed within a Research Electronic Data Capture (REDCap) database. REDCap (Version 16.0.31) is a secure software platform designed for data collection in research studies [32,33]. Once enrolled into the program, participants completed intake forms. DHCT staff assessed and verified the completed forms, then added the data to the DHCT REDCap database. Intake information instruments used in this study included:

1. An enrollment form, which gathered demographic information;
2. A self-efficacy rating scale;
3. A has/needs form to document items and services that the client had, needed, or did not need.

When participants were discharged from DHCT, additional information was collected, including the date of discharge and whether the participant no longer met the federal definition of homelessness and was therefore considered housed upon discharge.

### 2.4. Measures

#### 2.4.1. Independent Variables

Criteria for including variables in the study were that (1) we had reason to believe the variable may be associated with a difference in likelihood of being housed, based on prior literature or—specifically for unmet ICA needs, for which there was no prior literature—our own hypotheses, (2) there were no or few missing data points, and (3) the data could be interpreted meaningfully.

Demographic factors such as age, gender, and race were included because the literature has shown that these variables impact the likelihood of individuals becoming homeless and are therefore important considerations for interventions [19,34,35,36]. Education was also included for its known association with the likelihood of someone becoming homeless [37].

Age was utilized as a continuous variable. Gender was designated as male or female. One DHCT participant who identified as transgender was excluded from the study as they were the only person with this characteristic. Due to some race groups consisting of only a few individuals of that race, race was designated as (1) White or (2) not White for the analysis. The not White category included: Black or African American and more than one race. No respondents indicated Hispanic/Latino ethnicity. Likewise, due to there being only a few individuals in some educational attainment groups, education was categorized into (1) less than or equal to high school education, (2) obtaining a high school diploma or GED, or (3) more than high school education, which included some college, associate’s degree, bachelor’s degree, or graduate degree.

Perceived self-efficacy was measured via administration of the New General Self-Efficacy Scale [38]. This scale has been widely applied in studies on individuals in public housing developments [39], as well as various other contexts across different demographics and occupations [40,41,42,43]. The scale comprises eight questions assessing a self-efficacious mindset. Participants were able to respond with “strongly disagree” (one point), “disagree” (two points), “neither agree nor disagree” (three points), “agree” (four points), or “strongly agree (five points). Items were summed to obtain a self-efficacy score of five to 40, with 40 indicating the greatest perceived self-efficacy. Because this scale has only been validated for participants who have answered all eight questions [44], participants who had not answered all eight questions were excluded from our study.

The unmet ICA needs score comprises information from the has/needs form and includes a simple sum of five potential unmet ICA needs, including photo ID, birth certificate, social security card, cell phone, and email address. Of note, this is not a validated scale, but a set of identity and communication needs identified by program developers, all of which are definitively needed to obtain housing. The sum was 0 if the participant expressed no unmet ICA needs; the highest possible score was five, scored if a participant expressed a need for all of these items.

A participant was designated as not needing mental healthcare if “not applicable” or “has mental healthcare” was designated on the has/needs form, and as needing mental healthcare if “needs mental healthcare” was designated on the has/needs form. It was not clarified whether “needs mental healthcare” reflects untreated need, lack of access, active symptoms, or another explanation. Also, this study only evaluated mental healthcare need as a factor on enrollment in DHCT; mental healthcare need during or after enrollment was not studied.

#### 2.4.2. Outcome Variable

The outcome variable was dichotomized as being housed at discharge or not. A participant was considered to have an outcome of being housed if they were discharged to a setting that is not included in the federal definition of homelessness. The federal definition of homelessness includes four categories: (1) literally homeless, which also includes living in a shelter or becoming discharged from an institution from which they were homeless prior to entering, (2) at imminent risk of becoming homeless, with loss of residency within 14 days, (3) homeless under other federal statutes, (4) fleeing or attempting to flee domestic violence [45]. The outcome of “housed” included people who were lease holders on an apartment, were paying rent at a family care home or sober house, or who were housed in an assisted living arrangement. Return to homelessness after rehousing was not evaluated in our study.

### 2.5. Analysis

#### 2.5.1. Overview

Data were downloaded into an Excel spreadsheet from the REDCap database, then transferred from the Excel spreadsheet into a statistical analysis database. The data analysis for this paper was generated using SAS/STAT software, Version 9.4 of the SAS System for Windows. Copyright © 2002–2012 SAS Institute Inc.

#### 2.5.2. Univariate Analyses

Univariate statistics, including frequency and percentages, were calculated for all enrollment demographic, unmet ICA need, mental healthcare need, self-efficacy, and housing outcome variables. For ratio data, means and standard deviations were also calculated.

#### 2.5.3. Bivariate Analyses

Bivariate analysis was completed among all variables, and *p*-values were obtained to test the likelihood of correlation between variables being equal to zero. Significance was assessed at *p* < 0.1 and *p* < 0.05. For relationships including binary and continuous variables, *p*-values were based on Spearman’s test due to a non-normal distribution, consistent with prior studies on PEH [46]. A correlation coefficient of >|0.15| was designated as a strong correlation due to the sample size. The threshold for possible collinearity was determined to be a correlation coefficient > |0.7| for a statistically significant bivariate relationship.

For relationships including a non-binary categorical variable (education) and a binary variable, Chi square *p*-values were generally used; however, for the relationship between education and gender, Fisher’s exact *p*-value was used, as one cell size was less than five. For these associations, a Cochran–Armitage trend test was also performed to test whether the trends in distribution of the proportions for education categories were different for the categories in other variables.

#### 2.5.4. Multivariable Regression Models

Prior research notes the importance of controlling for confounding variables, which has been shown to, when accounted for, explain results for studies on PEH in impactful ways [47]. Multivariable logistic regression models against the outcome of housed were created using a conceptual framework of prior relationships among the variables based on causal logic and prior research, the results of the bivariate analysis, and temporality. By temporality, we mean the sense that age, race, and gender are definitive from birth (even if gender differs from sex at birth); education is attainable later; and perceived self-efficacy, mental health status, and meeting of ICA needs at point of entry into the program develop. A logistic regression approach was used. The first model included age, race, and gender as predictors of being housed. The second model included these variables, as well as education. The third model included all variables in the study. The fourth model included the variables that were significant in models 1–3. The use of multiple models allowed exploration of relationships among predictor variables as related to outcome. *p*-values and odds ratios with corresponding Wald confidence limits were generated. Significance was assessed at *p* < 0.1 and *p* < 0.05.

## 3. Results

### 3.1. Univariate Statistics

Of the 142 participants in the study, the mean age was 51.5 years, with a range of 22.1 years to 78.1 years. The majority were male (81.0%); most were Black or African American (68.3%). In total, 34.5% of participants had less than high school level education, 33.1% had high school education or a GED, and 32.4% had more than high school level education. Mental healthcare was not applicable for 16.9% of participants, 9.9% of participants had mental healthcare, and 73.2% of participants needed mental healthcare. The average self-efficacy score was 28.1 out of 40, with a range of 12 to 40. The average unmet ICA need score was 1.7, with a range of 0 to 5.0. The majority had an outcome of being housed (61.3%) (Table 1).

Table 1 shows descriptive statistics of DHCT participants.

### 3.2. Bivariate Analyses

#### 3.2.1. Overview

Only one independent variable—race—had a statistically significant association with the outcome of being housed in the bivariate analysis. A number of independent variables had statistically significant associations with each other.

#### 3.2.2. Spearman Correlations

The relationship between race and the outcome of being housed was significant with a *p*-value of 0.064 and a correlation coefficient > |0.15|. Among the independent variables, bivariate relationships which were significant at *p* < 0.05 and with relatively high positive correlation coefficients > |0.15| included that females were more likely to be White, higher educational attainment was associated with higher self-efficacy scores, and needing mental healthcare was associated with more unmet ICA needs; statistically significant relationships with relatively high negative correlation coefficients included that older age was associated with lower self-efficacy scores, higher educational attainment was associated with fewer unmet ICA needs, and higher self-efficacy score was associated with fewer unmet ICA needs. One bivariate relationship was significant at *p* < 0.1 and had a relatively high correlation coefficient > |0.15|; this was the negative relationship between needing mental healthcare and having a lower self-efficacy score. One relationship—race and self-efficacy score (*p* < 0.001)—had a weak correlation coefficient paired with statistical significance.

#### 3.2.3. Chi Square or Fisher’s Exact Tests and Trend Tests

The association between education and gender was significant with females being more likely to have higher educational attainment (*p* = 0.006), as was the trend test of the distribution of the proportions for education categories over the gender categories (*p* = 0.002). The association between education and race was not significant, but the trend test was significant for the distribution of the proportions for education categories over race categories (*p* = 0.075) (Table 2).

Table 2 shows *p*-values and correlation coefficients or trend tests from the bivariate analysis. For relationships including binary and continuous variables, Spearman’s *p*-values were used, and Spearman’s correlation coefficients are shown below, in parentheses. For relationships including the categorical variable (education) and a binary variable, Chi square and Fisher’s exact *p*-values were used, and *p*-values from Cochran–Armitage trend tests are shown below, in parentheses.

### 3.3. Multivariable Regression Models

#### 3.3.1. Collinearity

Because the highest absolute value of a correlation coefficient between two independent variables in our analyses was 0.233, there was no concern about collinearity.

#### 3.3.2. Concordance

The concordance values for the models ranging from 0.57 to 0.68 show that our model was somewhat better at classifying outcomes than random chance.

#### 3.3.3. Model Output

In Models 1–3, statistical significance for independent variables was achieved only at the level of *p* < 0.1. In the Demographics Model (Model 1), race was a significant predictor of being housed; the odds of a White participant being housed were 1.995 times the odds of a Black participant being housed, 95% CI [0.892, 4.461]. With the addition of education to the model (Model 2), race remained a significant predictor of being housed, with a slight increase in the odds ratio; the odds of a White participant being housed were 2.018 times the odds of a Black participant being housed, 95% CI [0.892, 4.565]. The association between education and being housed was not statistically significant. In the Comprehensive Model, race remained a significant predictor, with another small increase in the odds ratio. Two of the three newly added variables—self-efficacy score, and unmet needs—were also statistically significant predictors of being housed. The odds of a White participant being housed were 2.048 times the odds of a Black participant being housed, 95% CI [0.884, 4.741]. The odds of a participant being housed were 0.938 times the odds for a participant with a self-efficacy score lower by one point, 95% CI [0.875, 1.005]. The odds of a participant with one more unmet ICA need being housed were 0.761 times the odds of a participant with one less unmet ICA need being housed, 95% CI [0.575, 1.006].

In the Parsimonious Model, race, self-efficacy score, and unmet ICA needs remained significant predictors of being housed, and race achieved statistical significance at *p* = 0.043. Again, the odds ratio increased a small amount for the race variable. The odds of a White participant being housed were 2.188 times the odds of a Black participant being housed, 95% CI [0.970, 4.934]. The change in the odds ratio for self-efficacy was negligible (from 0.938 to 0.937, 95% CI [0.875, 1.005], [0.878, 1.000], respectively) and the odds ratio for unmet ICA needs was unchanged (0.761, 0.761, 95% CI [0.575, 1.006], [0.584, 0.992], respectively).

The odds ratio indicating the likelihood of White PEH being more likely to be rehoused than non-White PEH exhibited only minimal change with the stepwise addition of other variables in our models; therefore, this difference in likelihood of being rehoused is not caused by the influence of other variables included in our study, but rather, something else (Table 3).

Table 3 shows odds ratios and Wald confidence limits from regression model results for predicting the outcome of being housed.

## 4. Discussion

This study evaluated the association between demographic factors and other characteristics at time of enrollment and the outcome of being housed at discharge for PEH enrolled in a transitional care program. Our results suggested that the likelihood of PEH being rehoused is associated with race, perceived self-efficacy score (albeit with a negligible effect size), and unmet ICA need burden.

### 4.1. Race

Notably, the majority of the literature, excluding one study [12], throughout the last ten years has found that Black PEH are less likely to be rehoused than White PEH [17,18,19,48], despite the increase in efforts to address homelessness and reform rehousing processes [49]. There are numerous factors that could be perpetuating this disparity; one in particular could be what some studies have shown regarding the impacts of racism and discrimination in the process of rehousing PEH [50,51]. Perhaps institutional racism at the level of the general transitional care program itself or the landlord or leasing agency could be impacting this difference; racial disparities in familial financial support, illness severity, disability burden, trauma exposure, criminal legal system interactions, and employment inequities could also be contributing. Our findings imply that structural barriers affecting non-White unhoused individuals likely persist even when utilizing supportive services, reflecting potential inequities within the rehousing process itself, indicating a need for programs’ success metrics to include equity outcomes. Further exploration is necessary to understand what exactly drives the difference in the likelihood of rehousing and to discover what other variables may be contributing to the lower likelihood of non-White PEH being rehoused; examining mechanisms underlying this disparity, such as housing discrimination, criminal legal system record barriers, chronic mental or medical illness, and family composition could be valuable to explore. With a greater understanding and awareness around these racial differences in rehousing PEH, more effective and targeted housing interventions could be created and employed to more equitably rehouse PEH [18]. For example, programs could start developing and implementing standardized vulnerability scoring tools (as opposed to discretionary prioritization), auditing rehousing outcomes by race regularly, setting measurable equity targets, requiring documentation from programs and landlords/leasing agencies for denial of rehousing of individuals to reduce bias in rehousing and/or partnering with job placement programs that actively partner with employers with fair hiring practices.

### 4.2. Unmet ICA Needs

A novel contribution to the literature from this study is that having more unmet ICA needs, specifically with regard to the five ICA needs included in our study, makes PEH less likely to be rehoused. While not otherwise directly addressed in relation to likelihood of rehousing in the previous literature, this finding supports the existing literature noting the importance of identification documentation [30] and access to cell phones [31] in rehousing processes. More studies are needed to evaluate the role of ICA needs in association with the likelihood of PEH being rehoused across other programs and populations. If the evidence continues to support the importance of meeting ICA needs, then it would be appropriate for transitional care programs and other interventions for PEH to prioritize meeting these needs more effectively so as to increase the likelihood of PEH being rehoused.

This supports the notion that up until now, research on PEH has focused on individual characteristics, demographics, and health and/or mental health status; there has been much less investigation into concrete objectives—such as addressing unmet ICA needs—that social services could be very efficacious at addressing while PEH are in rehousing processes. By way of example, a recent study advocates for the development of a validated, standardized tool to measure health and general care needs for PEH. The authors of the study note that at present there is only one validated tool that assesses multiple domains to plan services for PEH, and this tool is only applicable in clinical settings [52]. That tool, like many others, focuses on housing, employment, and health, but does not inquire about ICA needs or other similarly straightforward needs faced by PEH. Our data support the necessity and potential impact of assessing a wide variety of needs of PEH, including ICA needs, in order to prompt development of interventions to meet those needs, which our data indicates will impact the likelihood of rehousing PEH. This implies the need for a standardized tool inquiring about ICA needs and highlights a future research direction about the role of different ICA needs in rehousing.

### 4.3. Self-Efficacy Score

Contrary to one remote study, we did not find that PEH with lower self-perceived self-efficacy have a decreased likelihood of exiting homelessness [13], but rather that there was essentially no difference in the odds of PEH being rehoused when comparing a self-efficacy score one point higher or lower. While the association between self-efficacy and being housed was statistically significant, the odds ratio was so close to one as to make the association of no practical significance. While there is very little literature about self-efficacy specifically, a recent study about resilience—a close psychological concept to self-efficacy—identifies specific barriers to resilience for PEH. The authors argue that there is a need for systemic efforts to address identified barriers to psychological resilience [53], supporting the general need for more research on PEH’s self-efficacy and related psychological concepts.

### 4.4. Other Variables

Although other studies have found that being female [13,14] or having higher education [11,12] may make PEH more likely to be rehoused, the findings in this study—that neither of these variables impact likelihood of rehousing—do not concur. Our findings that age and mental healthcare need did not impact likelihood of rehousing contribute to the existing mixed literature on the role of age [12,15,16] and access to mental health services [25,26,27,28] for PEH with mental illness.

### 4.5. Limitations

Some limitations of the study are the limited diversity of our sample along the lines of race and gender; there were only a few race categories included, which were combined into the non-White group, and one participant had to be excluded from the study due to being the only participant that identified as transgender. Also, seeing as 45 of 187 enrolled participants were excluded, there is a risk of selection bias. We used both *p* < 0.1 and *p* < 0.05 as thresholds for significance; in this observational study with a relatively small sample size, using *p* < 0.1 increases the risk of Type I error. Numerous bivariate correlations are tested; with multiple comparisons, the chance of a false positive increases. Additionally, this was a small study including only one program; however, this is the first time the variable of unmet ICA needs, a critical actionable variable, has been evaluated. Further, the specific context of DHCT could impact the results of this study; perhaps regional variation in related services, demographics, or yet unidentified aspects of this specific program could impact the findings of the study. On this note, one study found that of young unhoused adults in the United States, 32% did not complete high school or obtain a GED, 53% had a high school diploma or GED, and 15% had some postsecondary education; our distribution had a higher proportion of individuals with more than a high school diploma or GED, a lower proportion of individuals with a high school diploma or GED, and a similar proportion of individuals without a high school diploma or GED. There could be a difference in the distribution of educational attainment from our study population to the general unhoused population, which could impact generalizability and applicability of the study [37]. Lastly, there is evidence that there are biases in self-reporting mental health conditions and treatment for some vulnerable populations [54]; this could have affected our study results.

Nonetheless, the generalizability limitation is true of most studies of this type. The study had concordance of key findings with prior literature and shed light on the importance of testing a new variable of unmet ICA needs, the significance of which in this study has clear, actionable implications for transitional care programs.

## 5. Conclusions

This study demonstrated that non-White PEH are less likely to be rehoused than White PEH, highlighting a historical association that continues to exist despite efforts to reduce disparities in rehousing of non-White and White PEH. Additionally, having more unmet identification and communication access needs makes PEH less likely to be rehoused. These results contribute to the understanding of the demographics and other characteristics contributing to the likelihood of PEH being rehoused after engagement with transitional care programs. Our findings have important implications for public health policy, suggesting that further understanding around racial inequity in rehousing PEH could allow for the development of more effective and targeted housing interventions to achieve equitable rehousing. Also, increased understanding around the importance of meeting identification and communication access needs for PEH being rehoused could highlight a need for increased social service efforts to address these needs. Although this study was limited by its limited diversity of participants and inclusion of only one program, it offers a foundation for future research to investigate demographic factors, previously studied characteristics, and a newly defined variable of unmet ICA needs and their impact on the likelihood of PEH to be rehoused after engagement with a transitional care program.

## Figures and Tables

**Table 1:** Descriptive statistics n = 142.

Variable	Statistic	
Age	Mean (SD)	51.5 (9.7)
	Min, Median, Max	22.1, 52.9, 78.1
Sex	N (%)	
Female		27 (19.0)
Male		115 (81.0)
Race	N (%)	
White		41 (28.9)
Black or African American		97 (68.3)
More than one		4 (2.8)
Education	N (%)	
<high school		49 (34.5)
high school or GED		47 (33.1)
>high school		46 (32.4)
Self-Efficacy	Mean (SD)	28.1 (5.7)
	Min, Median, Max	12.0, 19.0, 40.0
Mental Healthcare	N (%)	
Not applicable		24 (16.9)
Has		14 (9.9)
Needs		104 (73.2)
Unmet ICA Needs	Mean (SD)	1.7 (1.4)
	Min, Median, Max	0.0, 1.0, 5.0
Housed	N (%)	
Yes		87 (61.3)
No		55 (38.7)

**Table 2:** Bivariate analysis.

	Age	Gender, Female	Race, White	Education	Self-Efficacy Score	Mental Healthcare, Needs	Unmet ICA Needs
Age							
Gender, female	0.363 (0.077)						
Race, white	0.906 (−0.010)	0.048 ** (0.167)					
Education	0.365 (0.077)	0.006 ** (0.002) **	0.203 (0.075) *				
Self-efficacy score	0.035 ** (−0.177)	0.435 (0.060)	<0.001 ** (0.100)	0.005 ** (0.233)			
Mental healthcare, needs	0.812 (0.121)	0.557 (−0.050)	0.400 (0.071)	0.400 (0.178)	0.056 * (−0.161)		
Unmet ICA needs	0.183 (0.112)	0.185 (0.112)	0.789 (0.023)	0.044 ** (−0.170)	0.011 ** (−0.214)	0.011 ** (0.213)	
Housed	0.856 (−0.015)	0.284 (0.091)	0.064 * (0.156)	0.287 (0.688)	0.163 (0.118)	0.150 (0.121)	0.198 (0.098)

* indicates significance at *p* < 0.1. ** indicates significance at *p* < 0.05.

**Table 3:** Logistic regression models.

	Demographics			Demographics + Education			Comprehensive			Parsimonious		
Concordance	0.57			0.61			0.68			0.63		
	OR	95% CI	*p*	OR	95% CI	*p*	OR	95% CI	*p*	OR	95% CI	*p*
Age	1.007	0.972–1.044	0.696	1.008	0.972–1.046	0.664	1.003	0.965–1.043	0.872			
Gender, female	1.485	0.585–3.770	0.405	1.469	0.562–3.840	0.433	1.334	0.489–3.642	0.573			
Race, white	1.995	0.892–4.461	0.093 *	2.018	0.892–4.565	0.092 *	2.048	0.884–4.741	0.094 *	2.188	0.970–4.934	0.059 *
Education < high school, vs. high school or GED				0.578	0.245–1.364	0.521	0.526	0.215–1.287	0.447			
Education > high school, vs. high school or GED				0.541	0.224–1.306	0.379	0.505	0.196–1.298	0.385			
Self-efficacy score							0.938	0.875–1.005	0.070 *	0.937	0.878–1.000	0.051 *
Mental healthcare, needs							0.581	0.244–1.385	0.221			
Unmet ICA needs							0.761	0.575–1.006	0.055 *	0.761	0.584–0.992	0.043 **

* indicates significance at *p* < 0.1. ** indicates significance at *p* < 0.05.

## Data Availability

The original contributions presented in this study are included in the article. Further inquiries can be directed to the corresponding author.

## Abbreviations

The following abbreviations are used in this manuscript:

PEH	People experiencing homelessness
DHCT	Durham Homeless Care Transitions
ICA	Identity and communication access
